# Cellular Senescence in Diabetes Mellitus: Distinct Senotherapeutic Strategies for Adipose Tissue and Pancreatic β Cells

**DOI:** 10.3389/fendo.2022.869414

**Published:** 2022-03-31

**Authors:** Takaaki Murakami, Nobuya Inagaki, Hiroshi Kondoh

**Affiliations:** ^1^ Department of Diabetes, Endocrinology and Nutrition, Kyoto University Graduate School of Medicine, Kyoto, Japan; ^2^ Geriatric Unit, Graduate School of Medicine, Kyoto University, Kyoto, Japan

**Keywords:** adipose tissue, aging, cellular senescence, diabetes mellitus, obesity, pancreatic β cell, senolysis, tailored senotherapy

## Abstract

Increased insulin resistance and impaired insulin secretion are significant characteristics manifested by patients with type 2 diabetes mellitus (T2DM). The degree and extent of these two features in T2DM vary among races and individuals. Insulin resistance is accelerated by obesity and is accompanied by accumulation of dysfunctional adipose tissues. In addition, dysfunction of pancreatic β-cells impairs insulin secretion. T2DM is significantly affected by aging, as the β-cell mass diminishes with age. Moreover, both obesity and hyperglycemia-related metabolic changes in developing diabetes are associated with accumulation of senescent cells in multiple organs, that is, organismal aging. Cellular senescence is defined as a state of irreversible cell cycle arrest with concomitant functional decline. It is caused by telomere shortening or senescence-inducing stress. Senescent cells secrete proinflammatory cytokines and chemokines, which is designated as the senescence-associated secretory phenotype (SASP), and this has a negative impact on adipose tissues and pancreatic β-cells. Recent advances in aging research have suggested that senolysis, the removal of senescent cells, can be a promising therapeutic approach to prevent or improve aging-related diseases, including diabetes. The attenuation of a SASP may be beneficial, although the pathophysiological involvement of cellular senescence in diabetes is not fully understood. In the clinical application of senotherapy, tissue-context-dependent senescent cells are increasingly being recognized as an issue to be solved. Recent studies have observed highly heterogenic and complex senescent cell populations that serve distinct roles among tissues, various stages of disease, and different ages. For example, in high-fat-diet induced diabetes with obesity, mouse adipose tissues display accumulation of *p21* ^Cip1^-highly-expressing (*p21* ^high^) cells in the early stage, followed by increases in both *p21* ^high^ and *p16* ^INK4a^-highly-expressing (*p16* ^high^) cells in the late stage. Interestingly, elimination of *p21* ^high^ cells in visceral adipose tissue can prevent or improve insulin resistance in mice with obesity, while *p16* ^high^ cell clearance is less effective in alleviating insulin resistance. Importantly, in immune-deficient mice transplanted with fat from obese patients, dasatinib plus quercetin, a senolytic cocktail that reduces the number of both *p21* ^high^ and *p16* ^high^ cells, improves both glucose tolerance and insulin resistance. On the other hand, in pancreatic β cells, *p16* ^high^ cells become increasingly predominant with age and development of diabetes. Consistently, elimination of *p16* ^high^ cells in mice improves both glucose tolerance and glucose-induced insulin secretion. Moreover, a senolytic compound, the anti-Bcl-2 inhibitor ABT263 reduces *p16* ^INK4a^ expression in islets and restores glucose tolerance in mice when combined with insulin receptor antagonist S961 treatment. In addition, efficacy of senotherapy in targeting mouse pancreatic β cells has been validated not only in T2DM, but also in type 1 diabetes mellitus. Indeed, in non-obese diabetic mice, treatment with anti-Bcl-2 inhibitors, such as ABT199, eliminates senescent pancreatic β cells, resulting in prevention of diabetes mellitus. These findings clearly indicate that features of diabetes are partly determined by which or where senescent cells reside *in vivo*, as adipose tissues and pancreatic β cells are responsible for insulin resistance and insulin secretion, respectively. In this review, we summarize recent advances in understanding cellular senescence in adipose tissues and pancreatic β cells in diabetes. We review the different potential molecular targets and distinctive senotherapeutic strategies in adipose tissues and pancreatic β cells. We propose the novel concept of a dual-target tailored approach in senotherapy against diabetes.

## Introduction

Diabetes mellitus is a growing issue of public health worldwide. In 2021, the estimated prevalence of patients with diabetes mellitus reached 537 million, and diabetes mellitus was responsible for 6.7 million deaths (1). The number of patients is predicted to increase further to 643 and 783 million by 2030 and 2045, respectively (1). In addition, the majority of individuals with type 2 diabetes mellitus (T2DM) are over 65 years of age. Actually, it is estimated that almost 1 in 4 people have T2DM, and that the number is still increasing (1, 2). To resolve this problem, it is essential to understand the detailed pathogenesis of diabetes and to develop a new strategy for treatment from the viewpoint of clinical and molecular aging.

Increased insulin resistance and impaired insulin secretion have central roles in the pathophysiology of T2DM (3). Insulin resistance is caused by impaired responses to insulin signaling in peripheral tissues such as muscle, liver, and fat. Insulin resistance is accelerated by obesity accompanied by accumulation of dysfunctional adipose tissues, followed by a compensatory increase in insulin secretion (4, 5). Subsequently, declines in pancreatic β-cell function develops diabetes mellitus in susceptible subjects (4). Amounts and functions of adipose tissue are affected by aging as well as by calorie intake, physical activity, and other aspects of health status (5, 6). Thus, it is well known that aging is associated with increased insulin resistance, suggesting an important potential contribution of cellular senescence in adipose tissue that affects the onset and progression of the disease (6).

It is also known that insulin secretion decreases with aging (7, 8). Systemic evaluation of insulin secretion is affected not only by the ability of individual β cells to secrete insulin, but also by the β-cell mass (BCM) (3, 9). Age-dependent decline in insulin secretion has been reported in studies using isolated human islets (10, 11). Although it is assumed that BCM is not affected by aging alone (9, 12), BCM can be changed dramatically during the onset and progression of obesity and diabetes, and decline of BCM can heavily influence the status of diabetes, such as glycemic control and responses to anti-diabetes treatment (3, 9, 13, 14). To meet increased demand for insulin secretion under increased insulin resistance, compensating BCM expands. Such expansion may be limited partly due to the decrease in β-cell proliferation with aging (15, 16). Age-associated changes in β-cell functions and proliferation suggest the importance of senescent aspects of β cells in pathogenesis of T2DM. Therefore, a deeper understanding of cellular senescence in β cells, as well as adipose tissue can offer novel therapeutic targets for the disease.

Human aging is accompanied by complex symptoms and disease states, including diabetes and obesity, due to progressively declining functions of various tissues and organs during aging. Strehler proposed four major properties of organismal aging: universality, intrinsicality, progressiveness, and deleteriousness (17). Consistent with his idea of universality, it has been widely accepted that senescent cells accumulate *in vivo* in aged tissues and organs (18). During organismal aging or the progression of diseases, normal cells and tissues *in vivo* are subjected to various stresses, causing cellular damage, necessitating repair, adaptation, apoptosis, or various defense responses. Senescence is a cellular state characterized by irreversible cell cycle arrest with functional decline due to telomere shortening or senescence-inducing stresses, e.g., deoxyribonucleic acid damage, oncogenic stress, and oxidative stress. Distinct from non-senescent cells, these senescent cells display several characteristic features, including enlargement with flattened morphology, change in nuclear structure, formation of foci expressing H2Aγ, increased expression of cell cycle inhibitors (p16^Ink4^ and p21^Cip1^), and others (18).

*INK4/ARF* locus encodes for the tumor suppressor; p16^Ink4^ and p19^Arf^. Both p16^Ink4^ and p19^Arf^ function as cell cycle inhibitors, by inhibiting cyclin-dependent kinase (CDK)4/6 and preventing p53 degradation, respectively (19). p21^Cip1^, another CDK inhibitor, is one of transcriptional targets of p53. *INK4/ARF* locus is epigenetically regulated in normal cells. In young cells, this locus is silenced by the polycomb repressive complexes PRC1 and 2, including chromobox protein homolog (CBX)7, B cell-specific Moloney murine leukemia virus integration site (BMI)1 and enhancer of zeste homolog 2, whose disruption downregulates repressive epigenetic marks of trimethyl histone H3K27 on the locus (20). In senescent cells, epigenetic activation of *INK4/ARF* locus induce irreversible cell cycle arrest, while ectopic expression of CBX7 or BMI1 bypass senescence in primary cells. In addition, several epigenetic fatcors (Mixed lineage leukemia protein-1, Jumonji domain-containing protein-3, or Zuotin-related factor 1) are also involved in these regulations (21).

However, irreversible progression due to aging is partly questioned. For example, telomere length is one of the well-known markers of cellular senescence *in vitro*. Telomeres are shortened after replicative exhaustion. New technology to assess the telomere length in leukocytes revealed that telomere length is closely linked to patient status in relation to cancer, atherosclerosis, heart failure, diabetes, depression, and chronic inflammation (22–24). Telomere shortening is partly reversed by changes in life style, including physical exercise, diet without calorie excess, relaxation, and so on (25, 26). These findings suggest that part of the aging phenotype is reversible.

Descriptions of deleterious aspects of aging are now controversial because of the double-edged sword of the behavior of senescent cells. Even young cells with intact telomeres suffer a senescent state under oncogenic insult, e.g., activation of oncogenic Ras, designated as oncogene-induced senescence (OIS) (27). Cells in OIS are also observed *in vivo* and are thought to serve as a defense against malignant progression of tumors (28). On the other hand, senescent cells also show enhanced secretion of proinflammatory cytokines and chemokines with activation of the nuclear factor-kappa B (NF-kB) pathway (29), resulting in what is known as a senescence-associated secretory phenotype (SASP) (30). Moreover, activation of NF-kB pathway renders senescent cells resistant to apoptotic stimuli due to upregulation of anti-apoptotic factors, such as X-linked inhibitor of apoptosis protein and Bcl-2 (29). A SASP provokes chronic inflammation, which promotes development of aging-related diseases. Thus, senescent cells accumulate *in vivo* not only in aged tissues, but also in organ dysfunction resulting from various lifestyle diseases, which plays a central role the pathogenesis (18).

Based on these ideas, either abrogation of chronic inflammation or elimination of senescent cells is emerging as a potential therapeutic strategy for aging-relevant diseases. Antibody therapy for inflammatory cytokines, e.g., interleukin (IL)-6 and tumor necrosis factor (TNF)-α, has already been clinically applied using molecularly targeted drugs. Recently, an anti-IL-1 antibody drug (canakinumab) was developed for treatment of some autoimmune diseases. Interestingly, the Canakinumab Anti-inflammatory Thrombosis Outcome Study (CANTOS) revealed that canakinumab prevented atherosclerosis and lung cancer among about 10,000 elderly people (31). This is an epoch-making example of a successful outcome by removal of chronic inflammation. Moreover, recent advances in aging research have suggested that removal of senescent cells, known as senolysis, is a promising approach (senotherapy) to reduce chronic inflammation and to treat aging-related diseases.

In this review, we summarize recent advances in understanding cellular senescence in adipose tissues and pancreatic β cells in diabetes. We review different potential molecular targets and distinctive senotherapeutic strategies in adipose tissues and pancreatic β cells. Consequently, we propose the novel concept of a dual-target tailored approach in senotherapy against diabetes.

## Emergence and Development of Senotherapy

In globally aging societies, numbers of patients with multiple aging-relevant diseases are increasing. For example, a person diagnosed as hypertensive and diabetic with atherosclerosis may simultaneously be treated by another physician for osteoporosis and dementia, and all of these conditions are promoted or exacerbated by aging. Thus, medical costs for combinations of disease-specific drugs for their illnesses are increased, creating a serious burden for aging societies (32). If targeting “aging” itself is feasible to treat multiple diseases, such burdens could be reduced. As research targeting “cellular senescence/chronic inflammation” is developing rapidly, removal of senescent cells, called “senolysis”, is a potentially attractive approach (33). This proposal is based on the novel concept that deleterious aspects of organismal aging can be delayed or prevented by removing senescent cells, the source of chronic inflammation (33).

**Figure 1:**
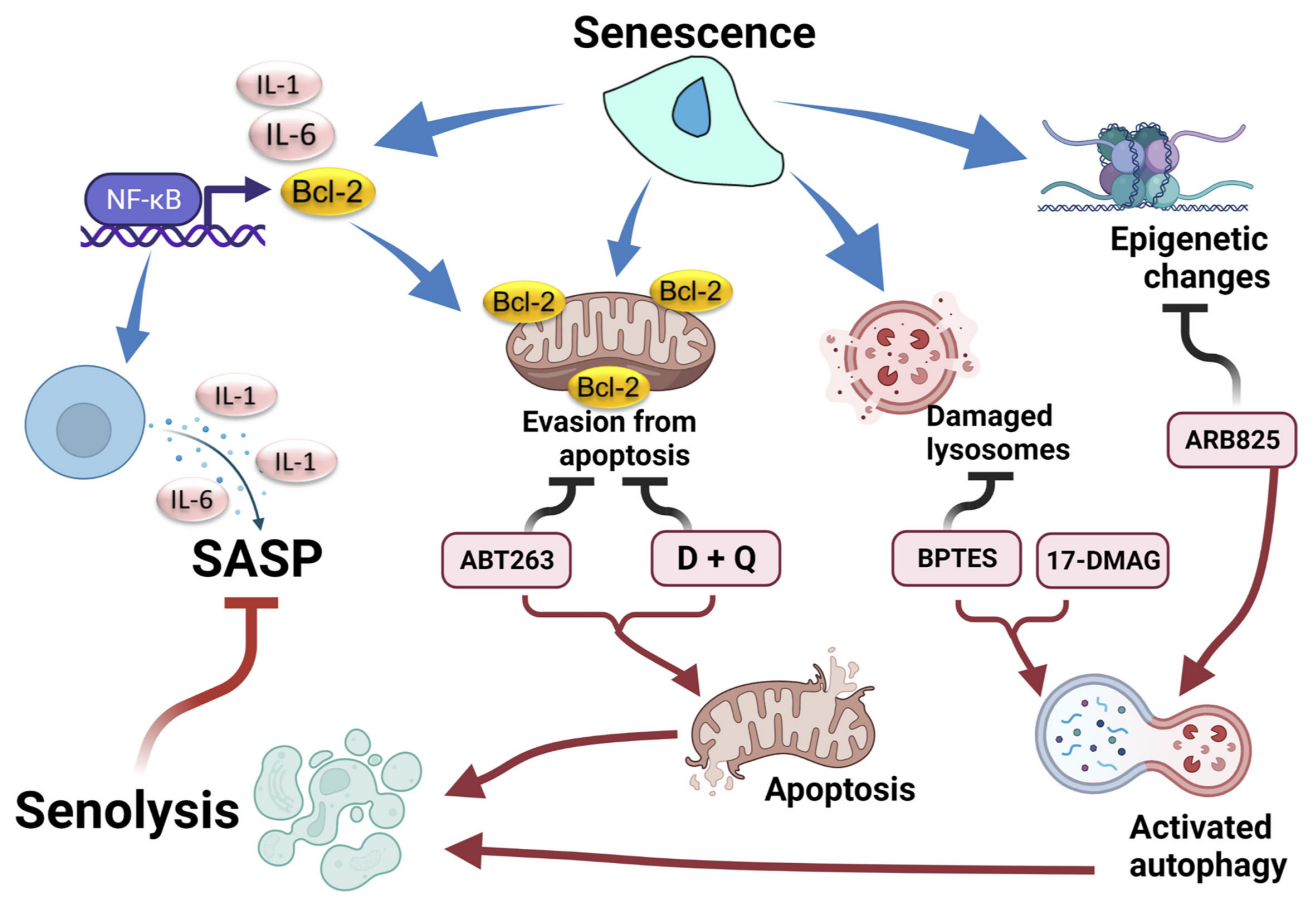
Senolytic response after chemically targeting senescent properties. Senescent cells display several properties: senescence-associated secretory phenotype (SASP) by activation of the NF-κB pathway, evasion of apoptosis by activation of the Bcl-2 family, damage to lysosomes by an acidic environment, and epigenetic modulation. These properties are counteracted by senolytic drugs, followed by suppression of SASP. BTES, GLS inhibitor. 17-DMAG, inhibitor for HSP. ARB825, epigenetic modulator. Created with Biorender.

Earlier work observed that the aging phenotype in progeria mice with a hypomorphic mutation in *BubR1*, a checkpoint gene, was partly restored by *p16* ^Ink4^ deletion, but not by ablation of *p19* ^Arf^, which results in inactivation of the *p53*/*p21* ^Cip1^ pathway (34), and that genetic removal of *p16*-high cells has been established as an efficient form of potential senotherapy (35). Later, the anti-apoptosis gene *Bcl-2* inhibitor (ABT263, navitoclax) was shown to eliminate senescent cells (**Figure 1**) (33). ABT263 was originally developed as an anticancer agent. However, the anti-Bcl-2 inhibitory effect of ABT263 was also effective for targeting aging cells with anti-apoptosis properties. ABT263 effectively improves aging-relevant symptoms in model mice, including cognitive impairment or atherosclerosis. However, precautions should be taken in regard to future application of senotherapy for human diseases. As some senolytic compounds are re-purposed anticancer drugs, profound side-effects cannot be overlooked. For example, ABT263 treatment provokes transient thrombocytopenia and neutropenia (36). Moreover, elimination of *p16*-high vascular endothelial cells disrupts blood-tissue barriers in mouse livers (37).

Subsequently, in addition to anti-Bcl-2 inhibitors, including ABT263, candidate drugs for senolysis have being reported (**Table 1** and **Figure 1**). The combination therapy of dasatinib plus quercetin (D+Q) exerted a senolytic effect by inhibiting several kinases and was effective in treatment of various age-related diseases such as chronic renal disorder, diabetes mellitus, and pulmonary fibrosis (38). Foxo4-DRI was developed as a peptide that inhibited binding of p53 and Foxo4 (40). Foxo4-DRI treatment induced p53-dependent senolysis and improved phenotypes in the Frail model (XpdTTD/TTD mouse). HSP90 is important for maintenance of proteostasis. HSP90 inhibitors were also effective in improving frailty with senolysis (41). ARB825, an inhibitor of the BET family epigenetic regulator, effectively removes aged cells and suppresses progression of liver cancer (42). Furthermore, an inhibitor of GLS1, a metabolic enzyme, exerted a senolysis effect on aging cells, resulting in improvement of various aging dysfunctions (43). Thus, targets of senotherapy now include mechanisms involving cell cycle control, anti-apoptosis, autophagy, proteostasis, environmental or metabolism homeostasis, and epigenetics (**Table 1**) (36).

**Table 1:** Senolytic compounds and their molecular targets.

Senolytic compounds	Molecular targets/pathways	*In-vivo* effective senescent cell types/tissues	References
Dasatinib	Src kinase/tyrosine kinase	D+Q; Mouse p16^high^ and p21^high^ cells in WAT, pancreatic β cells	(38, 39)
Quercetin	Bcl-2 family, p53/p21, PI3K/Akt signaling		(38, 39)
Foxo-DRI	Binding of p53 and Foxo4	Mouse liver and kidney	(40)
Alvespimycin (17-DMAG)	HSP90	Liver and kidney of progeroid Ercc1^−/Δ^ mice	(41)
ARV825	BET family	Mouse hepatic stellate cells	(42)
BPTES	GLS1	Mouse kidney, lung, liver, and adipose tissue	(43)
ABT263 (Navitoclax)	Bcl-2 family	Mouse p16^high^ cells in islets	(33, 39)
ABT199 (Venetoclax)	Bcl-2	Pancreatic β cells of NOD mice	(44)
ABT737	Bcl-2 family	Pancreatic β cells of NOD mice	(44)

D+Q, dasatinib and quercetin; WAT, white adipose tissue; DRI, D-retro inverso; HSP90, heat shock protein 90; Ercc1, excision repair cross-complementation group 1; MEF, murine embryonic fibroblast; BET, bromodomain and extraterminal domain; BPTES, bis-2-(5-phenyl- acetamido-1;3;4-thiadiazol-2-yl)ethyl sulfide; GLS1, glutaminase 1; NOD mice, non-obese diabetic mice.

## Cellular Senescence in Adipose Tissues

During aging, several changes in adipose tissues are observed, affecting both the quantitative and qualitative profiles (45). While fat mass in the body increases due to increased cell counts and hypertrophy of adipocytes since the early old stage (46), aging-dependent decline of adipogenesis is induced due to dysfunctional progenitor cells (47). Moreover, sterile inflammation, called as chronic inflammation, is frequently accompanied with adipose tissues in aged and obese individuals (47). All these changes in adipose tissues during aging provide significant impact on insulin sensitivity, followed by development of diabetic condition.

In various organs, senescence of white adipose tissue (WAT) is well characterized, and is called fat senescence (48). The earliest observation of accumulation of senescent cells was in WAT and lung tissue of aged mice with a *p16*-promoter reporter (49). Similarly, senescent cells were shown to accumulate in WAT of progeria mice, obese mice (50), aged and obese human (47). Strikingly, WAT transplantation from progeria or obese mice to control mice provoked diabetes with impaired insulin resistance, implying that fat senescence is partly responsible for the onset of diabetes (51).

Aged preadipocytes display impaired adipogenesis with increased inflammatory response (52), while aged adipocyte also provokes inflammatory response through SASP (45). Thus, cellular senescence in WAT exerts causal effect on the pathogenetic processes. Accumulation of both p16^Ink4^ and p21^Cip1^ are observed during fat senescence. In progeria models, e.g. mice with *BubR1* hypomorphic alleles, p53/p21 axis plays protective role for fat senescence (53). In contrast, tissue-specific ablation of p53 alleviate insulin resistance in obese or forth generation of *Tert*-deficient mice, implicating the beneficial effect of p53 on fat senescence (51). On the other hand, earlier work observed that the aging phenotypes in progeria mice, including fat senescence, were partly improved by p16^Ink4^ deletion, but not by p19^Arf^ inactivation (34). Thus, both positive and negative aspects of p53 are observed in fat senescence.

## Senotherapeutic Targets for Adipose Tissue in Diabetes

**Figure 2:**
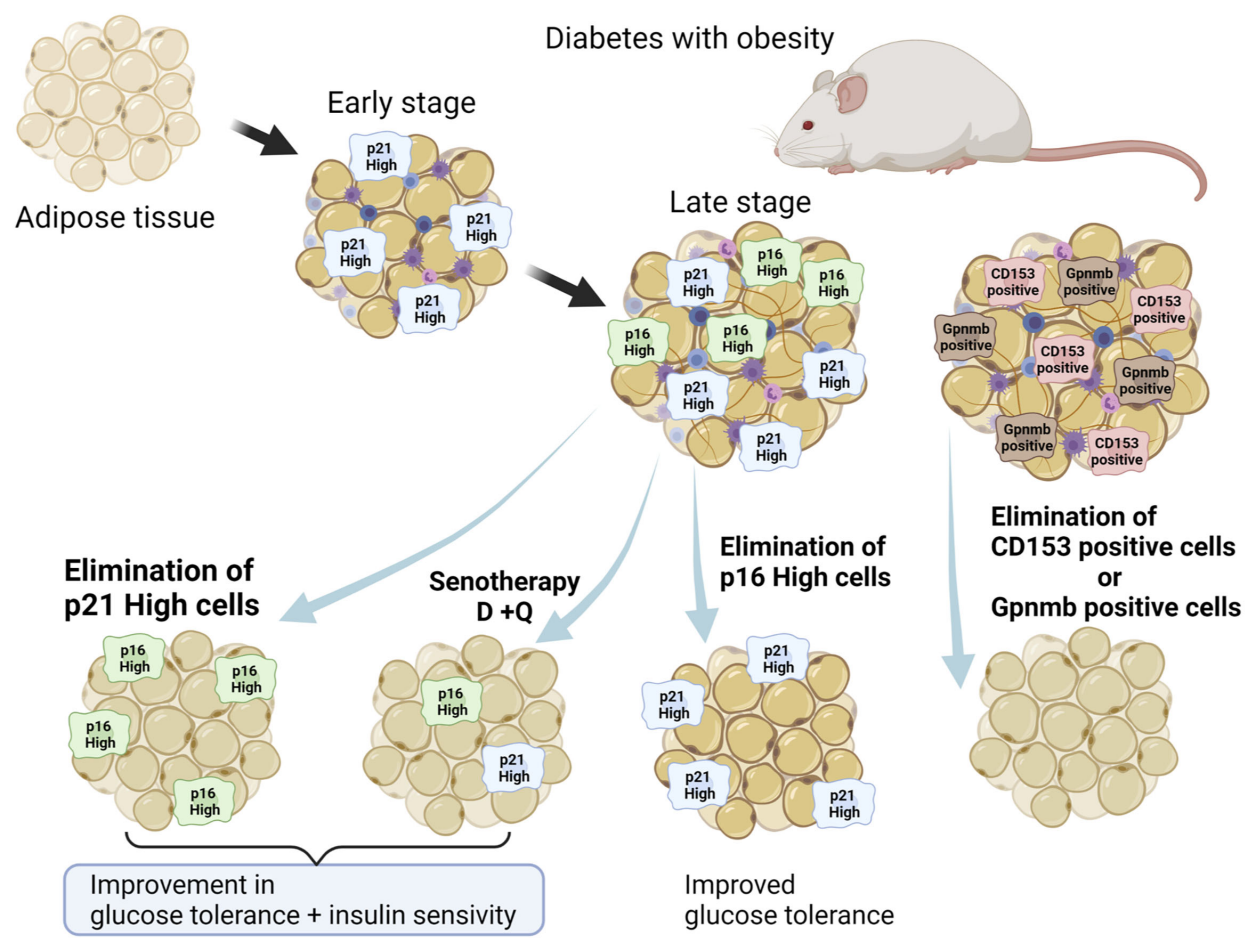
Senolytic approach to fat senescence. Several types of senescent cells reside in WAT in obesity: p16-high cells, p21-high cells, GPNMB-high cells, and CD153 positive immune cells. Elimination of such senescent cells in WAT alleviates symptoms of diabetes with obesity. Created with Biorender.

Not only genetic removal of *p16*-high cells (35), but also chemical senotherapy with D+Q alleviated insulin resistance by terminating senescent cells in WAT (38) (**Figure 2**). In addition to senescent adipocytes, senescent T cells that were CD153 positive invaded and resided in senescent WAT, resulting in enhanced chronic inflammation due to SASP (54). Elimination of senescent T cells that were CD153 positive in WAT also improved diabetes (55), suggesting that SASP in WAT is one of critical factors for its pathogenesis.

Recently, Wang et al. reported the pathological significance of senescent cells manifesting elevated expression of *p21* ^Cip1^ (*p21* ^high^) in murine WAT, apart from p16-high cells (56). Both p21-high and *p16* ^Ink4^-high-expressing (*p16* ^high^) cells in WAT share senescence-like features. However, in WAT of obese mice, *p21* ^high^ cells, but not *p16* ^high^ cells, accumulated during the early stage of a high-fat diet (HFD)-feeding regimen (two months), while both were observed later (10 months). On the other hand, p16-high cells reside predominantly in the aged pancreas. Interestingly, recent findings suggested a significant role for p21^Cip1^ in SASP, called the p21-activated secretory phenotype (PASP) (57). PASP functions as a marker for early immunosurveillance as the macrophage-attracting chemokine C-X-C motif chemokine ligand (CXCL) 14 is produced in *p21* ^high^ cells, not in *p16* ^high^ cells. In addition, a recent study identified glycoprotein nonmetastatic melanoma protein B (GPNMB) as a molecular target for senolytic therapy (58). Analysis of transcriptomic data from senescent vascular endothelial cells revealed that GPNMB was a molecule with a transmembrane domain that was enriched in senescent cells (seno-antigen). Genetic ablation of *Gpnmb*-positive cells attenuated senescence in adipose tissue and improved systemic metabolic abnormalities in mice that were fed a HFD (58). Thus, fat senescence composes different subclasses of senescent cells, including *p16* ^high^ cells, *p21* ^high^ cells, *Gpnmb* ^high^ cells, and CD153 positive immune cells (**Figure 2**). Moreover, the outcome of senotherapy in WAT differs between elimination of *p16* ^high^ and *p21* ^high^ senescent cells, while D+Q eliminated both *p16* ^high^ and *p21* ^high^ cells (38). While elimination of *p21* ^high^ cells in WAT suppressed SASP phenotypes and improved both glucose tolerance and insulin sensitivity, senolysis targeting of *p16* ^high^ cells to alleviate insulin resistance was less efficient.

Thus, p21^Cip1^ is a potential candidate for senotherapy in human diseases, such as obesity-coupled diabetes, given that *p21* ^high^ cells are responsible for NF-kB dependent inflammation (56). However, the beneficial impact of p21^Cip1^ deletion on aging-related events has been controversial (59). This is partly due to the fact that unlike p16^Ink4^, p21^Cip1^ has significant physiological functions *in vivo* (60). Indeed, it has been accepted that SASP and senescent cells themselves also serve physiologically important functions in embryonic development, wound healing, tissue repair, and tumor-suppression (36). Therefore, careful consideration should be given to when and to which organs *p21* ^high^ senolysis should be applied in order to minimize these complications.

## Cellular Senescence of Pancreatic β Cells in Aging

Similar to adipose tissue, senescence of pancreatic β cells has been investigated. According to evaluations of islet glucose-stimulated insulin secretion, including static and perfusion systems (10, 11), oral glucose tolerance test (61, 62), HOMA-β (63), intravenous glucose tolerance test (64, 65), and euglycemic insulin clamp (66), age-dependent functional decline of human β cells has been consistently reported, separate from insulin resistance. Even under various types of stimulation and metabolic demands for β-cell proliferation, age-dependent decline of β cell proliferation has been reported in rodents and humans (15, 16, 67–69). A certain subpopulation of β cells expresses senescence-associated β-galactosidase activity (SA-β-Gal), p16^INK4a^, p53BP1, and insulin-like growth factor 1 receptor (IGF1R) at early stage, whose levels increases with age (70). Recent studies have revealed that changes in functions and proliferation of aged β cells *in vivo* resemble those observed in senescent β cells *in vitro*, which may contribute to development of diabetes (70). Senescent β cells, in which SA-β-Gal, p16^INK4a^, p53BP1, and IGF1R are highly expressed, display impaired function with increased basal insulin, which is similar to β cells in aged subjects (70). Comparative RNA sequencing (RNA-seq) between SA-β-Gal^+^ and SA-β-Gal^−^ β cells in C57BL6/J male mice demonstrated increased expression of senescence markers (*p16* ^INK4a^, *p21* ^Cip1^) and SASP ones (*Il6*, *Il1a*, *Tnf*, *Ccl2*, and *Cxcl1*), down-regulation of β cell markers (*Ins1*, *Pdx1*, *Mafa*, *Nkx6.1*, and *Neurod1*), and up-regulation of genes that are usually suppressed in SA-β-Gal^−^ β cells (*catalase* and *ldha*), along with increased SA-β-Gal (39). Additionally, senescent β cells showed decreased expression of genes involved in glycolysis (*Gck*), cellular depolarization (ATP-dependent K^+^ channel, voltage-gated Ca^2+^ channels, chloride channels, cation channels, and sodium channels), incretin signal pathways (*Glp1r*, *Gcgr*), and components of insulin granules (*Slc30a8*, *ins1*, and *ins2*) (7). These transcriptional changes were similar to those observed during aging of β cells in mice expressing green fluorescent protein under mouse insulin promoter (MIP) (70). Other studies showed that a subpopulation of β cells in mice formed replicative senescence with transcriptional de-repression of cyclin-dependent kinase inhibitors, such as *p16* ^INK4^, *p15* ^INK4b^, and *p19* ^Arf^ during aging (71, 72), and a study showed increased expression of p27^KIP1^ and histone deacetylase (HDAC) 1 in aged rat β cells (73). Therefore, senescent β cells have unique transcriptional changes that can be associated with the decline of functions and proliferations of β cells with aging.

In addition to transcriptional analyses, SASP factors are produced and secreted by senescent β cells. Isolated senescent β cells (SA-β-Gal^+^) secreted more SASP including IL-6, TNF, and CXCL1 than nonsenescent β cells (SA-β-Gal^−^) in C57BL6/J male mice (39). Interestingly, isolated mouse islets cultured in the presence of conditioned medium from SA-β-Gal^+^ β cells showed increased expression of *p16* ^INK4a^ compared with those in medium from SA-β-Gal^−^ β cells (39). These findings suggested that senescent β cells might contribute to senescence of neighboring β cells *via* secretion of SASP in a paracrine fashion. Although the composition of SASP factors varies among cell types (74), a proteomic analysis of conditioned media collected from mouse senescent (SA-β-Gal^+^) β cells revealed β-cell SASP signature proteins (75). Represented pathways included IL signaling, a6b1 and a6b4 integrin signaling, phosphatidylinositol-3 kinase (PI3K)-Akt signaling, neurotrophin signaling, cellular responses to growth factor stimuli, regulation of cellular responses to stress, and extracellular matrix remodeling. In addition, a proteomic analysis of conditioned media collected from human senescent (SA-β-Gal^+^) β cells showed that human β-cell SASP is enriched in factors associated with similar pathways such as cytokine and IL signals, cellular responses to growth factors, and mitogen-activated protein kinase activation, while human β-cell SASP shares only several common factors with murine SASP, that is, AIP, MFRP, PAK6, KLK6, HDAC8, GPC6, and IDE (75). Pathways related to inhibition of proliferation and negative cell differentiation are included unique to human β-cell SASP (75). Moreover, changes in SASP factors from senescent and non-senescent human β cells were very similar to transcriptional changes found in β cells from donors with and without T2DM (75). Inflammatory pathways induce chemotaxis of immune cells in the islets and impair GSIS of β cells *via* enhancement of endoplasmic reticulum (ER) stress (76–78). Thus, the conserved signaling pathways of SASP in senescent β-cell also may contribute to aging of β cells and the pathophysiology of T2DM.

## β Cell Senescence Under Metabolic Stress

Importantly, not only aging itself but also metabolic stress, insulin resistance in particular induces and accelerates senescence of β cells (6, 7). In C57BL6/J male mice, eight weeks of feeding a HFD increased the percentage of SA-β-Gal^+^ cells in islets as well as expression of aging and SASP-related genes in islets (39). Similarly, administration of an insulin receptor antagonist S961 induced acute, severe insulin resistance in C57BL6/J mice (79), which was accompanied by increased SA-β-Gal^+^ cells and increased mRNA levels of aging and SASP-related genes including *p16* ^INK4a^, *p21* ^Cip1^, and *Bambi* in islets (39, 70). Surprisingly, these changes under S961-induced insulin resistance are partially reversible. Expression of aging and SASP-related genes decreased and β-cell identity genes increased after improvement of insulin resistance with discontinuation of S961 administration (39). In addition, Ins2^Akita^ (Akita) mice, a model of chronic ER stress, showed an increased tendency for *p16* ^INK4a^ and *Bambi* mRNA expression (70). In humans, islets isolated from older donors contained more SA-β-Gal^+^ islet cells than those from younger ones, and those from donors with T2DM had more SA-β-Gal^+^ cells in islets (39). Moreover, higher expression of IGF1R was observed in donors with T2DM than in those without (39). Expression of p53BP1 in islets showed a correlation with body mass index in donors without T2DM (39). Taken together with these findings, metabolic stress such as insulin resistance may induce or accelerate senescence and aging of β cells.

Other than insulin resistance, stability of V-maf musculoaponeurotic fibrosarcoma oncogene homolog A (MafA) protein, which is a β cell-enriched transcription factor that promotes β-cell maturation and function, can also be associated with senescence of β cells (80). It is known that *MAFA* is a causative gene of maturity-onset diabetes of the young (MODY). Substitution of serine-64 with phenylalanine (S64F) in MAFA enhances the instability of MAFA protein and *MAFA* ^S64F^ male carriers can develop diabetes (81, 82). In male *MafA^S64F/+^* mice, islets displayed increased expression of aging and SASP-related genes and increased senescence markers, such as SA-β-Gal, p21^Cip1^, p53BP1, and H2Aγ (80). In the same line, human EndoC-βH2 cells expressing MAFA^S64F^ increased expression of SA-β-Gal, p21^Cip1^, and p53BP1 compared to those with MAFA^WT^, and produced SASP factors (80). Moreover, senescence and acquisition of SASP in β cells may also be a significant pathogenic component of type 1 diabetes mellitus (T1DM). Senescent β cells are more frequently observed in the pancreas of patients with islet autoantibody-positive recent onset T1DM, compared to individuals without diabetes or those who were islet autoantibody-positive prior to onset of T1DM (44). Similarly, in non-obese diabetic mice, a mouse model of T1DM, *p16* ^high^, and *p21* ^high^ cells in islets were increased and β cells acquired SASP during the progression of T1DM (44). These findings suggested a possible common pathophysiological role for β cell senescence across various etiologies of diabetes including MODY, T1DM, and T2DM, although further studies in other types of diabetes should be investigated.

Although a detailed time course of progression of β cell senescence remains to be clarified, β cell senescence may proceed through multiple stages. Although senescence procedures or markers may differ among tissues or cell types, the cell cycle arrest gene, *p21* ^Cip1^ (*Cdkn1a*) may be a candidate marker for early stage and/or an initial driver in β cell senescence, whereas another cell cycle arrest gene, *p16* ^INK4a^ (*Cdkn2a*) further maintains the cellular state (83, 84). Time-based observation using bleomycin-treated MIN6 cells demonstrated that *p21* ^Cip1^ mRNA expression increased substantially within five days after treatment, whereas *p16* ^INK4a^ expression increased by day 12 to a lesser extent (75). *p21* ^Cip1^ expression was correlated with expression of multiple mouse SASP factors such as *Gstp1*, *Gdf15*, *Dusp3*, and *Hsp90aa1*. Additionally, in S961-treated mice, single-cell RNA sequence analysis of islets showed that *p21* ^Cip1^ mRNA expression was increased compared with control mice and was reversed after discontinuation of S961 treatment (75). On the other hand, expression of *p16* ^INK4a^ increased in S961-treated mice and further increased after discontinuation of S961 treatment. expression of *p21* ^Cip1^ was correlated with up-regulation of aging and SASP-related genes and down-regulation of β-cell identity genes. Collectively, it was suggested that p21^Cip1^ may indicate entry into cellular senescence in β cells and lead to increased p16^INK4a^ as well as SASP factors, which can result in maintaining the cells in a senescence state (39, 75). However, it has also been reported that *p16* ^high^ cells accumulated rather than *p21* ^high^ cells in the pancreas of obese mice (39), which is largely different from proportions in adipose tissue (56). Actually, p16^INK4a^ can be a benchmark of β cell senescence. Transgenic mice overexpressing p16^INK4a^ displayed decreased islet proliferation and enhanced GSIS in islets, while p16^INK4a^ deficiency did not affect islet proliferation in young mice, but showed relative increases in old mice (71, 72). Therefore, the significance of p21^Cip1^ requires further validation as an early marker of β cell senescence, although p16^INK4a^ may have a pivotal role in pathogenesis of β cell senescence.

## Senotherapy Against Senescent Pancreatic β Cells in Diabetes

**Figure 3:**
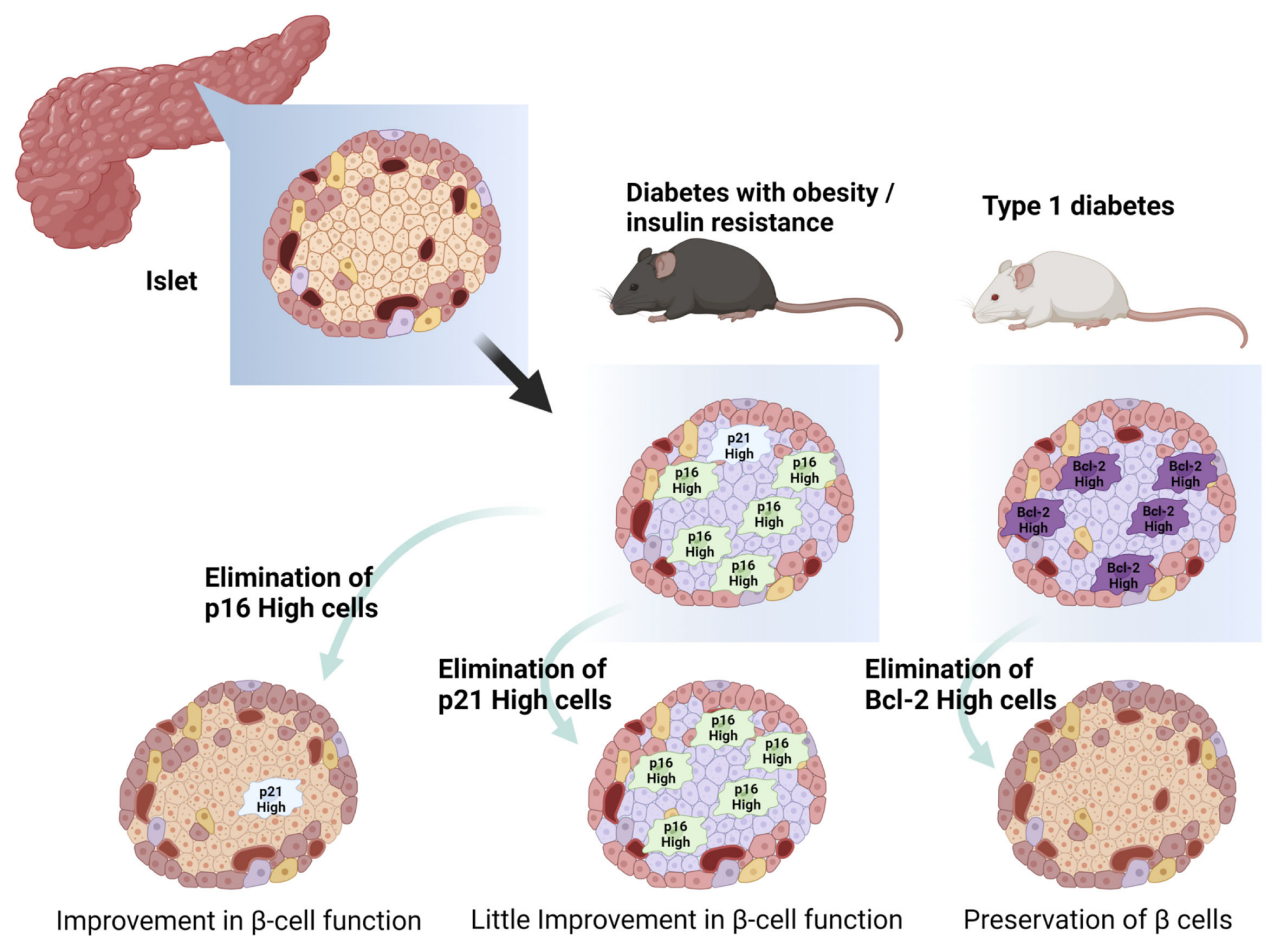
Senolytic approach to pancreatic β-cell senescence. p16-high cells, p21-high cells, Bcl-2 high cells reside in pancreatic β cells with diabetes. Elimination of such senescent cells in islets restores β-cell function and/or prevents destruction of β cells. Created with Biorender.

Based on the understanding of β cell senescence in development and progression of diabetes, the concept of senotherapy targeting senescent β cells has emerged recently (**Table 1** and **Figure 3**). Potential efficacy of senolysis on senescent β cells was brought from transgenic INK-apoptosis through targeted activation of caspase (ATTAC) mice, a whole-body FLAG-tagged transgenic that allows deletion of *p16* ^high^ cells on administration of B/B homodimerizer (35, 39). In aged INK-ATTAC mice, treatment with B/B homodimerizer improved β-cell function, which was shown as decreased basal insulin levels after fasting and improved GSIS *in vivo*, and it decreased expression of aging and SASP-related genes in islets (39). Additionally, B/B-treated INK-ATTAC mice under S961 administration also showed improved glucose tolerance and decreased expression of aging and SASP-related genes in islets, whereas those fed with HFD showed improved glucose tolerance and decreased expression of aging-related genes in islets. Consistently, ABT263 reduced *p16* ^high^ cells and unchanged *p21* ^high^ cells in islets, and restored glucose tolerance in INK-ATTAC male mice with S961 treatment (39). ABT263 treatment decreased the expression of SASP-related genes, but did not affect aging-related and β-cell identity genes. However, in INK-ATTAC female mice with HFD, ABT263 did not affect non-fasting blood glucose levels, although it reduced SA-β-Gal^+^ cells and expression of aging and SASP-related genes in islets. Differences between mice with S961 and HFD-induce insulin resistance may have occurred due to differences in senescence stages or their extent in the two models. In addition, other senolysis compounds, such as D+Q demonstrated senolysis efficacy on β cells *in vitro* whereas Q alone showed no effects on senescent SA-β-Gal^+^ β-cell mortality (39). These findings suggested that p16^INK4a^ is a potential candidate for senotherapy targeting β cells in T2DM. At the same time, interpretation of these results should be made carefully. Genetic clearance of *p16* ^high^ cells under HFD feeding did not improve insulin sensitivity significantly in an insulin tolerance test (39). Both the INK-ATTAC mouse model and ABT263 treatment cannot totally exclude the influence of other organs, including AT, liver, and muscle, on the restoration of glucose tolerance and β-cell senescence, since the INK-ATTAC mouse is a whole-body transgenic animal and ABT263 can be delivered systemically. Thus, the direct effect of senolysis on β cells should be further evaluated in additional studies using a β-cell specific INK-ATTAC animal model and/or *in vitro* assays of β-cell functions in islets composed of senescent and non-senescent β cells. Moreover, more suitable senolysis compounds should be explored for clinical applications of p16^INK4a^-targeted senotherapy in T2DM due to clinical intolerability, and they are seemingly less effective in HFD models with chronic insulin resistance of ABT263 (36, 37).

Another potential of senolysis in β cells has also been demonstrated in prevention or delay of T1DM onset. Bcl-2 inhibitors, including ABT199 (venetoclax), eliminated senescent β cells and reduced the incidence of diabetes in non-obese diabetic (NOD) mice (44). Islets of old NOD mice showed higher expression of Bcl-2, an anti-apoptotic pro-survival mediator, than young NOD mice or C57BL6/J mice of equivalent age (44). Bcl-2 inhibitors are mimetic compounds of the BH3 domain in the Bcl-2 protein family and can induce apoptosis in cells with a high level of Bcl-2 and related molecules (85). ABT737 inhibits Bcl-2 and other related molecules and ABT199 selectively inhibits Bcl-2 (86, 87). Treatment with ABT737 reduced *p16* ^INK4a^ and *p21* ^Cip1^ mRNA expression in islets of NOD mice *in vitro* and substantially decreased p16^INK4a^-expressing β cells in NOD mice *in vivo*. Treatment with ABT199 reduced secretion of SASP factors in cultured islets of NOD mice. Importantly, although treatment with ABT199 did not instantly affect glucose tolerance in NOD mice *in vivo* and GSIS in isolated islets from NOD mice *ex vivo*, it turned out later that short-term treatment with ABT199 prevented development of diabetes in NOD mice *in vivo* through reduction of islets with insulitis without any influence of β-cell proliferation (44). Although clinical evaluations of this therapeutic concept and efficacy of ABT199 in T1DM should be investigated further, this suggested that transient elimination of senescent or high Bcl-2-expressing cells may halt insulitis and preserve non-senescent islets, which lead to prevention or delay of T1DM onset.

## Conclusions and Future Perspectives

**Figure 4:**
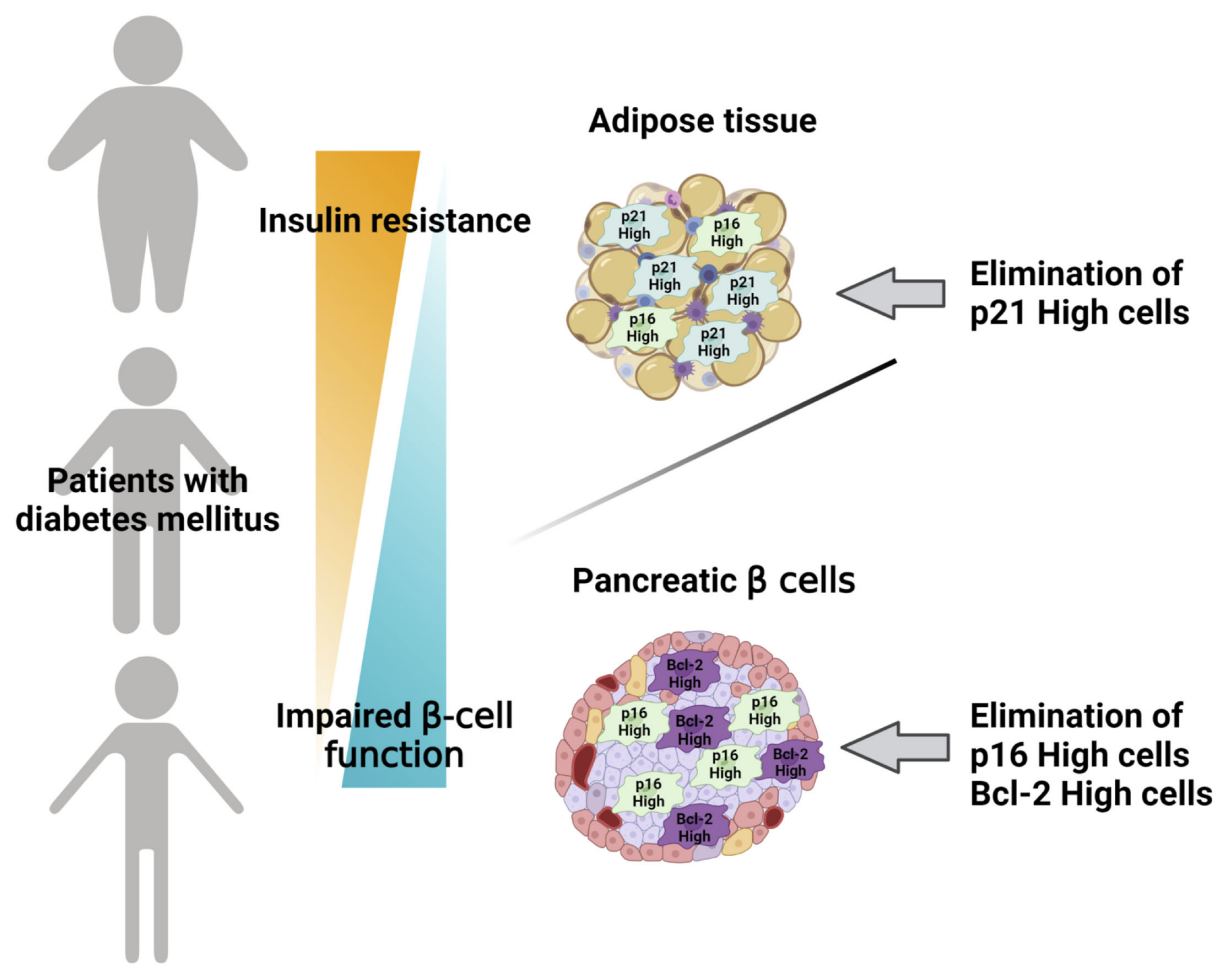
Dual-targeted approach in senotherapy for diabetes. A tailored strategy targeting adipose tissue and/or pancreatic β cells is proposed for senotherapy against diabetes. Distinctive senescence markers of adipose tissue and/or pancreatic β cells are targeted based on individual pathophysiological conditions of diabetes. Created with Biorender.

The last two decades of research have achieved remarkable advances in understanding of cellular senescence in adipose tissue and pancreatic β cells in diabetes, including their pathological role and contribution to development and progression of diabetes. Importantly, recent studies regarding senolysis have opened the possibility of senotherapy, a novel therapeutic concept, in diabetes. Since not only aging, but also metabolic stress, including insulin resistance, have had an impact on development, acceleration, and possible reversal of cellular senescence, individuals can harbor cellular senescence at different degrees even at the same age. Moreover, since potential distinctive markers and molecular mechanisms of cellular senescence have been discovered in adipose tissue and pancreatic β cells, degrees of cellular senescence and pathogenic contributions to diabetes can differ between these two major components of diabetes pathophysiology. In adipose tissue, p21^Cip1^ can be a hallmark of early-stage senescence and a main molecular target of senolysis to attenuate insulin resistance (**Figure 2**), whereas clearance of p16^INK4a^ can improve β cell functions and Bcl-2 can also be a molecular target of senolysis in pancreatic β cells, although clearance of *p21* ^high^ cells showed little effect on β cell functions (**Figure 3**). Therefore, a tailored approach targeting dual organs with distinctive molecular targets and therapeutic strategies in adipose tissue and pancreatic β cells should be examined as a novel senotherapeutic strategy against diabetes (**Figure 4**). Further investigations of molecular mechanisms of cellular senescence in diabetes and senolytic compounds with clinical efficacy and safety are warranted to realize a clinical application of this concept.

## Author Contributions

TM and HK performed a literature search, wrote, and edited the manuscript. NI reviewed and edited the manuscript. All authors contributed to the article and approved the submitted version.

## Conflict of Interest

NI received clinical commissioned/joint research grants from Daiichi Sankyo, Terumo, and Drawbridge Inc., speaker honoraria from Kowa, MSD, Astellas Pharma, Novo Nordisk Pharma, Ono Pharmaceutical, Nippon Boehringer Ingelheim, Takeda, Eli Lilly Japan, Sumitomo Dainippon Pharma, and Mitsubishi Tanabe Pharma, scholarship grants from Kissei Pharmaceutical, Sanofi, Daiichi Sankyo, Mitsubishi Tanabe Pharma, Takeda, Japan Tobacco, Kyowa Kirin, Sumitomo Dainippon Pharma, Astellas Pharma, MSD, Eli Lilly Japan, Ono Pharmaceutical, Sanwa Kagaku Kenkyusho, Nippon Boehringer Ingelheim, Novo Nordisk Pharma, Novartis Pharma, Teijin Pharma, and Life Scan Japan.

The remaining authors declare that the research was conducted in the absence of any commercial or financial relationships that could be construed as a potential conflict of interest.

## Publisher’s Note

All claims expressed in this article are solely those of the authors and do not necessarily represent those of their affiliated organizations, or those of the publisher, the editors and the reviewers. Any product that may be evaluated in this article, or claim that may be made by its manufacturer, is not guaranteed or endorsed by the publisher.
